# T Cell Receptor Engagement Triggers Its CD3ε and CD3ζ Subunits to Adopt a Compact, Locked Conformation

**DOI:** 10.1371/journal.pone.0001747

**Published:** 2008-03-05

**Authors:** Ruth M. Risueño, Wolfgang W. A. Schamel, Balbino Alarcón

**Affiliations:** 1 Centro de Biología Molecular Severo Ochoa, Consejo Superior de Investigaciones Científicas, Universidad Autónoma de Madrid, Madrid, Spain; 2 Max Planck-Institut für Immunbiologie, Freiburg, Germany; University Paris Sud, France

## Abstract

How the T cell antigen receptor (TCR) discriminates between molecularly related peptide/Major Histocompatibility Complex (pMHC) ligands and converts this information into different possible signaling outcomes is still not understood. One current model proposes that strong pMHC ligands, but not weak ones, induce a conformational change in the TCR. Evidence supporting this comes from a pull-down assay that detects ligand-induced binding of the TCR to the N-terminal SH3 domain of the adapter protein Nck, and also from studies with a neoepitope-specific antibody. Both methods rely on the exposure of a polyproline sequence in the CD3ε subunit of the TCR, and neither indicates whether the conformational change is transmitted to other CD3 subunits. Using a protease-sensitivity assay, we now show that the cytoplasmic tails of CD3ε and CD3ζ subunits become fully protected from degradation upon TCR triggering. These results suggest that the TCR conformational change is transmitted to the tails of CD3ε and CD3ζ, and perhaps all CD3 subunits. Furthermore, the resistance to protease digestion suggests that CD3 cytoplasmic tails adopt a compact structure in the triggered TCR. These results are consistent with a model in which transduction of the conformational change induced upon TCR triggering promotes condensation and shielding of the CD3 cytoplasmic tails.

## Introduction

Upon ligand binding, membrane receptors have to transmit information from their ectodomains to their cytoplasmic tails, and several mechanisms have been proposed to account for how this happens. One way would be to simply increase the local concentration of cytoplasmic tails by promoting aggregation of the ectodomains, which can be achieved by crosslinking with a multivalent ligand, or could even be induced by a monovalent ligand, if this binding promotes a conformational change which results in aggregation. The archetypal receptor aggregation mechanism is the activation of membrane receptor tyrosine kinases. Dimerization of these receptors results in activation of their cytoplasmic tyrosine kinase domains and auto-transphosphorylation of their tails [1], [2]. A second possible mechanism involves a conformational change in the cytoplasmic tails themselves, somehow transmitted from the ectodomains. The clearest example of this is provided by G protein-coupled receptors [3], [4]. The aggregation and conformational-change mechanisms are not mutually exclusive; indeed it has been suggested that membrane protein tyrosine kinase receptors also undergo a conformational change [5]. To date, much evidence has been gathered which strongly suggests that several receptors of immunological interest undergo a conformational change upon ligand binding. These include the erythropoietin receptor, the tumor necrosis factor receptors, Fas, the interleukin-6 receptor, and the B cell receptor [6]–[10]. In multichain membrane receptors the conformational change induced upon ligand binding could be a result of rearrangement of the quaternary structure of the complex.

The T cell receptor (TCR) complex is composed of ligand-binding subunits (TCRα and TCRβ) and signal transducing subunits (CD3γ,CD3δ,CD3ε and CD3ζ [CD247]) [11]–[13]. The ligand of the TCR consists of a peptide antigen bound to major histocompatibility complex (MHC) class I or class II molecules. Assembly studies, transfection and reconstitution experiments, and detergent dissociation studies suggest that the TCR complex components are organized as dimers [reviewed in [14]]. CD3ε forms non-covalently-bound alternate dimers with CD3γ and CD3δ, TCRα forms disulfide-linked dimers with TCRβ, and CD3ζ is expressed in the form of disulfide-linked homodimers.

In spite of significant advances in understanding how the TCR signal is propagated within the cell, little is known about the mechanisms that initiate TCR signaling. A number of models have been proposed, including oligomerization of the TCR complex [15], [16], conformational changes occurring within a single TCRα/β heterodimer or within the complete TCR complex [17], [18], geometrical rearrangements within a multivalent TCR complex [19], and segregation of tyrosine kinases and phosphatases from the TCR complex [20], [21]. The poor ability of monovalent (Fab) anti-CD3 antibodies to stimulate T cells compared with bivalent antibodies has long suggested ligand-induced oligomerization as a necessary component of the activation mechanism [22]–[24]. This model has been reinforced by experiments comparing monomeric and oligomeric forms of soluble ectodomains of MHC complexed with antigen peptide [25]–[27]. Nevertheless, there is growing evidence that TCR signaling also involves a conformational change. Early experiments showed that monovalent forms of certain clonotypic (anti-TCR) antibodies induce cocapping with CD4, in what has been taken as the first evidence of a ligand-induced conformational change in the TCR [28]. Further, the isolated tail of CD3ζ is converted from a phospholipid-bound helical form to a random coil upon tyrosine phosphorylation [29]. By using monovalent and multivalent TCR ligands, we have recently shown that a multivalent engagement is required for induction of the conformational change, and that TCR crosslinking and conformational change are both required for full T cell activation [30]. Indeed, the conformational changes associated with the induced fit of the complementarity-determining region loops have been proposed as a mechanism that contributes to ligand discrimination [31]. If conformational changes in the whole TCR complex are considered, and not only those in the complementarity-determining regions, this mechanism can be generalized to all TCR-pMHC interactions [32].

We have identified Nck as a ligand for the polyproline sequence (PPS) of CD3ε [17]. Within the TCR complex, CD3ε is in a non-binding state in non-stimulated T cells, but upon ligand (pMHC, superantigen or antibody) engagement, the CD3ε tail undergoes a conformational change that exposes the PPS for Nck binding. To understand the mechanisms that allow the transmission of the conformational change from TCR-complex ectodomains across the membrane to the CD3 cytoplasmic tails, we have now investigated whether the conformational change is transmitted to CD3 subunits other than CD3ε. Using a protease-sensitivity assay, we show that the tails of CD3ε and CD3ζ become protected from degradation upon induction of the conformational change. These results suggest that the conformational change in the TCR is transmitted to the tails, not only of CD3ε but of CD3ζ as well. In addition, our results suggest that contrary to our initial models [17], [33], the CD3 tails are not converted from a resting “closed” conformation into an active “open” conformation, but rather are converted from a loose, protease-sensitive conformation to a compact, protease-resistant conformation. We propose that both conformations be renamed as *loose* and *locked*, respectively.

## Results

### The CD3 tails adopt a compact conformation upon TCR engagement

We have previously reported that in a pull-down assay immobilized glutathione S-transferase (GST)-Nck binds to stimulated TCR complex but not to the non-stimulated complex [17]. Since Nck binds through its N-terminal SH3 domain to the PPS of CD3ε, the pull-down assay revealed a rearrangement of the CD3ε tail. The assay indicated that the non-stimulated TCR complex was in a non-binding conformation, which we called closed-CD3 [33]. This form was converted upon stimulation into a binding conformation, which we called open-CD3. This conformational change was also suggested by positive immunostaining of stimulated cells and tissues with the CD3ε's PPS-specific monoclonal antibody APA1/1 [34], [35]. Thus, the pull-down and APA1/1 staining assays both demonstrated that the PPS of CD3ε becomes exposed after TCR engagement.

**Figure 1:**
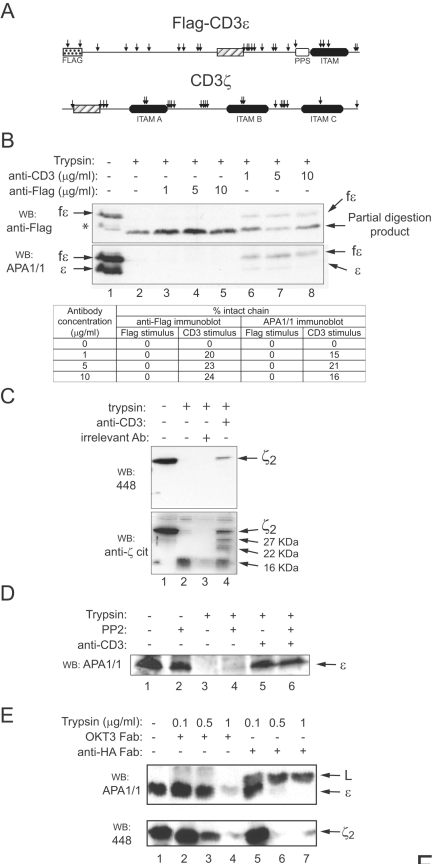
Effect of TCR ligation on the susceptibility of CD3ε and CD3ζ to limited proteolysis. (A) A cartoon of the CD3ε and CD3ζ subunits showing potential trypsin cleavage sites (arrows). Transmembrane domains are indicated with striped boxes, the polyproline sequence (PPS) with an open box, and the foreign Flag epitope, appended to the N-terminus of CD3ε, with a dotted square. The relative positions of the ITAMs within the cytoplasmic tails are marked. (B) Addition of an anti-CD3 antibody that induces TCR conformational change protects the tail of CD3ε from trypsin proteolysis. The indicated concentrations of anti-CD3 (OKT3) or anti-Flag antibody were added to a detergent lysate of Jurkat cells transfected with Flag-CD3ε before incubation with trypsin. The bands corresponding to CD3ε and its degradation products were detected by immunoblotting first with anti-Flag antibody and second with the PPS-specific antibody APA1/1. The asterisk indicates the presence of a protein fragment in lane 1 that corresponds to the partial proteolysis of Flag-CD3ε, probably caused by cellular proteases contained in the cell lysate. The balance sheet indicates the percentages of protected CD3ε molecules, calculated by densitometry of the anti-Flag and APA1/1 immunoblots. (C) The CD3ζ tails becomes protected from proteolytic cleavage after TCR triggering. A Jurkat cell lysate was incubated with 10 µg/ml of either OKT3 or an isotypic control antibody (OKT8) before digestion with trypsin. Immunoblotting was performed with anti-CD3ζ antibodies reacting with the C-terminus (448) or the whole tail (anti-ζcit). (D) Tyrosine phosphorylation does not affect the sensitivity of CD3ε to trypsin proteolysis. A Jurkat cell lysate was incubated with 10 µg/ml of OKT3 in the presence of 20 µM of the src kinase inhibitor PP2 for 15 min at room temperature, before digestion with trypsin. Immunoblotting was performed with antibody APA1/1. (E) Protection against trypsin proteolysis is independent of TCR aggregation. A Jurkat cell lysate was preincubated with 50 µg/ml of Fab fragments of OKT3 or the isotypic control antibody 12CA5 (anti-HA), before digestion for 15 min at 37°C with the indicated concentrations of trypsin. Immunoblotting was performed with APA1/1 followed by reprobing with the 448 antiserum. The presence of immunoglobulin light chain (L) from the anti-HA Fab fragment in the APA1/1 immunoblot is indicated.

To further define ligand-induced conformational change in TCR cytoplasmic tails, and also to study whether CD3 subunits other than CD3ε are affected, we performed a series of protease-sensitivity assays. Since the tail of CD3ε is rich in lysine and arginine residues that are recognized as cleavage sites by trypsin (Fig. 1A), we chose this protease for our studies. To allow examination of the products of C-terminal-end digestion, experiments were done in Jurkat T cells stably expressing a human CD3ε chain labeled at its N-terminus with a Flag epitope (fε-Jk cells). Trypsin digestion of detergent lysates of fε-Jk cells generated a CD3ε partial-digestion product of 19 kDa, representing a loss of 4 kDa, which is most of the CD3ε tail (Fig. 1B). The partial digestion product was not recognized on immunoblots by the PPS-specific antibody APA1/1, suggesting that this sequence had been degraded. Digestion was, however, partially inhibited by addition to the lysate of the stimulatory anti-CD3 antibody OKT3. In contrast, the anti-Flag antibody did not inhibit digestion. Anti-Flag stimulation of fε-Jk cells was previously demonstrated, using the pull-down assay, to be a poor inducer of the conformational change in the TCR, even though it induced tyrosine phosphorylation (Fig. S1). The OKT3-protected CD3ε band was recognized by APA1/1, suggesting that the PPS was protected (Fig. 1B). These results indicate that binding of a conformational-change-inducing antibody to the TCR complex renders the CD3ε resistant to trypsin digestion, providing further support that the tail of CD3ε undergoes a conformational change in response to engagement by a stimulatory antibody.

To determine whether the CD3ζ subunit also undergoes conformational change, trypsin-digested Jurkat T cell lysates were immunoblotted with antibody 448, which is specific for the C-terminal-most 34 amino acids of CD3ζ. This showed that the CD3ζ dimer was digested by trypsin upstream of the sequence recognized by the antibody (Fig. 1C). Incubation with the stimulatory antibody OKT3, but not with an irrelevant isotype-matched antibody, partially prevented the loss of the 448 epitope. Furthermore, reprobing the membrane with a polyclonal antibody raised against the whole tail of CD3ζ detected intermediate-sized partial-digestion products only in cell lysates that had been incubated with OKT3 (Fig. 1C, WB anti-ζcit).

Since TCR triggering with the anti-CD3 antibody OKT3 was performed after lysis of the cells in detergent, it was unlikely that the protective effect on the CD3ε and CD3ζ tails was due to shielding caused by tyrosine phosphorylation of the immune receptor tyrosine-based activation motifs (ITAMs) or to recruitment of signalling proteins such as ZAP70. Nevertheless, in order to exclude this possibility, we included the potent Src family kinase inhibitor PP2 [36] in the protease sensitivity assay. The result showed that, even in the presence of PP2, the triggered TCR acquired resistance to trypsin digestion (Fig. 1D), suggesting that the protection effect of TCR triggering was not due to a post-lysis modification of the TCR. Another important control was to demonstrate that the acquisition of resistance to trypsin was not due to the formation of aggregates that are poorly accessible to trypsin upon crosslinking of the TCR with the bivalent anti-CD3 antibody. Engagement of the TCR complex in Jurkat cell lysates with a monovalent Fab fragment of OKT3, but not the incubation with an isotype-matched irrelevant antibody, increased the resistance of the CD3ε and CD3ζ tails to trypsin digestion (Fig. 1E). This result indicates that the acquisition of resistance to trypsin digestion is not caused by aggregation of the TCR, and is consequent with previous evidence showing, with the pull-down assay, that a monovalent anti-CD3 antibody induces the conformational change [17]. The results shown in Figures 1D and 1E exclude alternate explanations, and suggest that the acquisition of resistance to trypsin digestion is caused by a conformational change transmitted to the CD3 tails.

**Figure 2:**
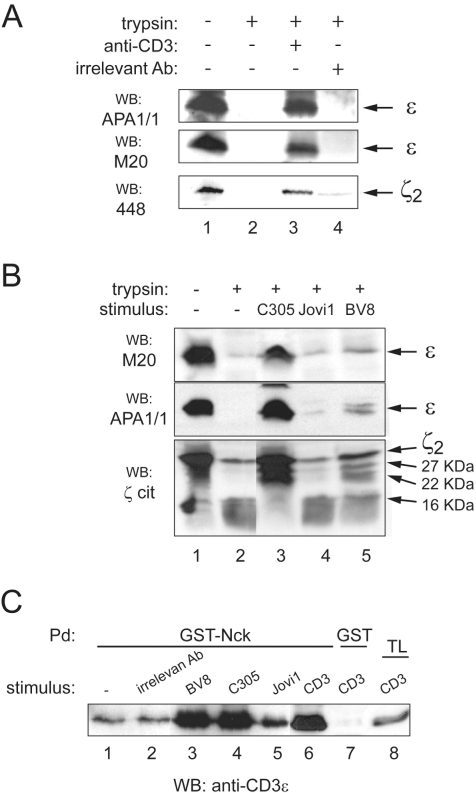
Stimulation of living T cells provokes the adoption of a protease-resistant conformation in CD3ε and CD3ζ. (A) CD3 ligation in living cells protects against proteolytic cleavage. Jurkat cells were unstimulated or stimulated for 5 min with 10 µg/ml of either anti-CD3 (OKT3) or an irrelevant antibody (HP2/6, anti-CD4). Detergent lysates were incubated with trypsin as in Fig. 1, and immunoblotting was performed with APA1/1, 448 and M20, an antibody specific for the C-terminal end of CD3ε. (B) Stimulation with certain anti-TCRα/β antibodies promotes the protease-resistant conformation in CD3ε and CD3ζ. Jurkat cells were stimulated for 5 min with the anti-Vβ antibodies BV8 (IgG2b) and C305 (IgM) or with the anti-Cβ antibody Jovi.1 (IgG1). Lysates were incubated with trypsin, and immunoblotting was performed as in A. (C) Differential capacity of the anti-TCRβ antibodies to induce the conformational change determined by the pull-down assay. Jurkat cells were stimulated with an irrelevant anti-CD4 antibody or with the indicated antibodies to the TCR complex, as in B. Lysates were subjected to pull-down with GST-SH3.1 (GST-Nck) or GST. The presence of the TCR in the precipitates was revealed by immunoblotting with anti-CD3ε (M20). TL, total cell lysate.

The acquisition of resistance to trypsin digestion by CD3ε and CD3ζ was also noted when OKT3 was used to stimulate intact Jurkat T cells before lysis, but not when an irrelevant antibody (anti-CD4) was used (Fig. 2A). Furthermore, the protected CD3ε and CD3ζ bands correspond to the full-length proteins, since they were recognized by M20 and 448, respectively, two polyclonal antibodies specific for the C-terminal ends. This result suggests that the conformational change involves a rearrangement of the whole tails.

The protective effect of TCR stimulation against trypsin digestion of CD3ε and CD3ζ tails was seen not only with anti-CD3 dimer antibodies (e.g. OKT3), but also with stimulatory antibodies for the TCRα/β heterodimer. Thus, stimulation of intact Jurkat cells with antibodies C305 and BV8, specific for the variable Vβ region, partly prevented the degradation of the CD3ε and CD3ζ tails by trypsin (Fig. 2B). This effect was, however, not seen with the anti-Cβ antibody Jovi.1, which is a poor inducer of the conformational change according to the GST-Nck pull-down assay ([17] and Fig. 2C). Isotypic differences cannot explain the differential effect of the anti-TCR antibodies, at least for antibodies OKT3, Jovi.1 and HP2/6 (irrelevant antibody in Fig. 2C, lane 2) which are of the same isotype (IgG2a). These results strongly suggest that TCR engagement induces a conformational change that is transmitted to the cytoplasmic tails of CD3ε and ζ.

**Figure 3:**
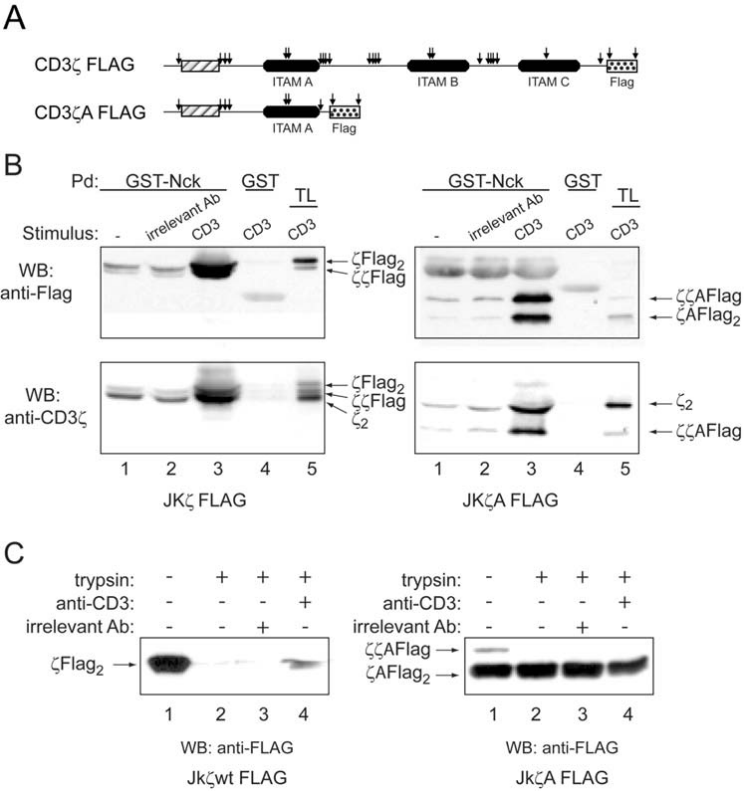
The membrane-proximal ITAM of CD3ζ is constitutively protected from limited trypsin digestion. (A) Cartoon showing the potential trypsin cleavage sites in CD3ζ and in a truncated CD3ζ lacking ITAMs B and C. The position of a Flag epitope appended to the C-terminal end of both constructs is indicated. (B) The presence of ζFlag or ζAFlag does not alter the ligation-induced conformational switch in the TCR. GST-SH3.1 pull-down was performed as in Fig. 2C with lysates of Jurkat cells transfected either with ζFlag (JkζFlag) or ζAFlag (JkζAFlag). (C) ITAM A is constitutively condensed. JkζFlag and JkζAFlag were stimulated with antibodies and the lysates digested with trypsin as in Fig. 2C. Immunoblotting was performed with anti-Flag antibody.

From the mobilities of the digestion products (Fig. 1A and 1C) it appeared that the cytoplasmic tail of CD3ε from non-triggered TCRs was completely digested, whereas the tail of CD3ζ was only partly accessible to trypsin. The estimated loss of relative mass in the CD3ζ_2_ dimer after digestion was 16 kDa (from 32 to 16 kDa), which is below the 24 kDa loss that would be expected if all the CD3ζ tail were digested (Supplemental material Fig. S2). Upon stimulation, partial proteolytic products were detected at 22 and 27 kDa (Fig. 1C). The sequences that separate the three Immune receptor Tyrosine-based Activation Motifs (ITAM) in CD3ζ are particularly rich in basic amino acids, and therefore in potential trypsin-cleavage sites (Fig. 1A, 3A). Inspection of ITAM distribution in CD3ζ suggests that the 27 kDa fragment could derive from a cleavage between ITAMs B and C, and the 22 kDa fragment from cleavage within ITAM B (Suppl. Fig. S2). The 16 kDa product resulting from digestion of CD3ζ in resting TCRs could derive from cleavage between ITAMs A and B. If these calculations are correct, they would indicate that compared with the more membrane-distal ITAMs, ITAM A might be constitutively protected from trypsin attack in the resting TCR. No antibody specific for ITAM A was available, so to test this we generated a truncated CD3ζ mutant with a Flag epitope appended immediately after ITAM A (Fig. 3A, construct ζAflag). Transfection of this construct into Jurkat cells generated disulfide-linked ζAflag homodimers and heterodimers of ζAflag and endogenous CD3ζ (Fig. 3B, total lysates, TL). TCR complexes containing either one (ζζAflag) or two ζAflag constructs (ζAflag2) underwent the conformational change after TCR triggering, as indicated by a positive reaction in the GST-Nck pull-down assay (Fig. 3B). However, ζAflag was completely resistant to trypsin digestion even in non-triggered TCRs, whereas the Flag epitope was completely digested when appended at the C-terminal end of full-length CD3ζ (Fig. 3C). These results suggest that the CD3ζ ITAM closest to the membrane is permanently protected from digestion and that the conformational change in the TCR modifies the exposure of the second and third ITAMs of CD3ζ to trypsin cleavage.[Fig pone-0001747-g004]

**Figure 4 pone-0001747-g004:**
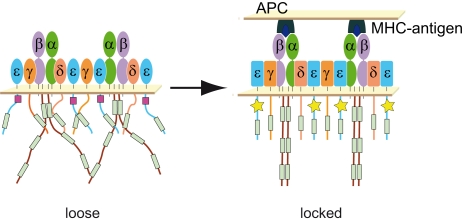
Model of conformational change in the cytoplasmic tails of CD3 subunits after TCR engagement. In non-stimulated cells, the CD3 subunits in the TCR are in a *loose* conformation that is accessible to trypsin digestion. The PPS of CD3ε (pink square) is in a non-binding conformation for Nck. After TCR engagement with pMHC or with stimulatory antibodies, the CD3 ectodomains adopt an active conformation (symbolized with rectangular forms) that is transmitted to the cytoplasmic tails of the CD3 subunits via a rotation and/or scissor movement. The cytoplasmic tails close up to form a compact structure that is less sensitive to proteolytic attack. This conformation permits Nck-binding by CD3ε.

## Discussion

Limited proteolysis has become an established tool for the study of conformational changes [37], [38]. For instance, the atrial natriuretic peptide receptor becomes susceptible to cleavage by exogenously added protease when bound to its lignad [38]. In this paper we have used limited proteolysis to study the induction of conformational changes in the TCR complex. Our results show that the cytoplasmic tails of CD3ε and CD3ζ subunits extracted from resting cells are almost completely digested by added trypsin, but become protected upon TCR triggering. Up to now, the strongest evidence for a conformational change in the TCR has come from studies showing ligand-induced exposure of the PPS in the cytoplasmic tail of CD3ε [17]. One biochemical assay is based on pull-down with immobilized GST-Nck, and has served to demonstrate that certain stimulatory anti-CD3 antibodies [17] and a panel of pMHC ligands [33] induce a conformational change in the TCR. A second assay to detect these conformational changes is based on exposure of a neo-epitope recognized by the monoclonal antibody APA1/1. This epitope coincides with the PPS in CD3ε and reveals, like the pull-down assay, a conformational change transmitted to the cytoplasmic tail of CD3ε [34]. Through the use of APA1/1 it has been possible to confirm that the TCR undergoes conformational changes during antigen recognition *in vivo*, and that the TCR complexes undergoing the conformational change are located in the immune synapse; these experiments moreover showed that the conformational change is elicited by full but not by partial agonist/antagonist peptides [34]. Unfortunately, the pull-down and APA1/1 recognition assays reflect the same molecular event (rearrangement of the PPS in the tail of CD3ε) and thus did not provide evidence of conformational change in TCR subunits other than CD3ε.

Previous evidence for conformational change in the cytoplasmic tail of CD3ζ was based on *in vitro* biochemical studies. Isolated CD3ζ cytoplasmic tails change from a lipid-bound helical structure to an unfolded conformation upon tyrosine phosphorylation [29]. These results support the earlier finding that a synthetic peptide corresponding to the third ITAM of CD3ζ adopts an α-helical structure in the non-phosphorylated form and a β strand conformation when phosphorylated [39]. However, both these studies were performed in cell-free systems with synthetic peptides and recombinant proteins, in the absence of other TCR subunits. The limited proteolysis studies reported in this paper therefore provide the first evidence that CD3ζ undergoes a conformational change within the TCR complex in response to ligand binding. Due to the lack of appropriate antibodies, we have not yet been able to study the effect of limited proteolysis on CD3γ and CD3δ, but a model could be proposed in which a conformational change is transmitted from the ligand-binding ectodomains of the TCRα/β to the cytoplasmic tails of all CD3 subunits.

The conformational change induced in CD3ζ, however, shows distinct features from those confirmed for CD3ε. Whereas the whole cytoplasmic tail of CD3ε is susceptible to proteolytic degradation in the non-triggered TCR and is protected upon stimulation, in CD3ζ ITAM A is protected even in non-triggered TCRs, and stimulation extends this protection to ITAMs B and C. Interestingly, CD3ζ is sequentially phosphorylated on its three ITAMs during T cell activation (for a review see [40]). In some cases ITAMs B and C are phosphorylated constitutively, producing the p21 tyrosine phosphorylated form of CD3ζ. In contrast ITAM A is phosphorylated only upon TCR triggering, yielding the p23 form. Although the detailed functional significance of the p21 and p23 forms of CD3ζ is not clear, it is well-established that phosphorylation of ITAM A defines the difference between triggered and non-triggered TCRs. In light of our limited proteolysis results, we suggest that, in a resting TCR, ITAM A is in a compact conformation that is not accessible to tyrosine phosphorylation by the priming src kinases [41], and that the conformational change not only compacts ITAMs B and C, but also reorients ITAM A into a conformation more susceptible to phosphorylation.

We recently described that both TCR crosslinking and conformational change are required for full tyrosine phosphorylation of different intracellular effectors [30]. In this regard, the acquisition of protease resistance by the tail of CD3ζ shown in the present study suggests that the conformational change may also affect the phosphorylation of CD3ζ and, therefore, the recruitment of ZAP70 and the subsequent phosphorylation of downstream effectors. This requirement of the conformational change for tyrosine phosphorylation appears to be contradicted by the activation of tyrosine phosphorylation with the anti-flag antibody, a poor inducer of the conformational change in Flag-CD3ε-expressing Jurkat cells (Supplemental Figure S1). Several explanations can be given. The most simple is that the anti-flag antibody induces the conformational change to a level that is sufficient to pass a threshold for activation of tyrosine kinases. A second possibility, is that TCR crosslinking in the absence of conformational change is sufficient for the activation of tyrosine kinases but not for a normal pattern of phosphorylation, i.e. TCR-associated tyrosine kinases could be activated without the TCR undergoing a conformational change, but the access to their potential substrates (ITAMs and downstream effectors) would be limited. Finally, the number of tools that we have to study the conformational change, or conformational changes, in the TCR is limited. The exposure of the PPS in CD3ε, revealed by the GST-Nck pull-down assay and APA1/1 epitope display [17], [34], and the trypsin sensitivity assay shown in the present study, might be only coarse methods to understand the fine tuning of signal transduction by the TCR.

The acquisition of resistance to trypsin digestion upon TCR stimulation suggests that, contrary to our prior prediction, stimulation does not shift the cytoplasmic tails of the TCR from a “closed” to an “open” conformation [17], [33]. Our present data indicate instead that the cytoplasmic tails in the non-engaged TCR are in a loose conformation that makes them accesible to trypsin digestion. We now prefer to name this conformation the Nck non-binding or *loose* conformation. Upon antibody stimulation, a conformational change is transmitted from the TCR ectodomains to the CD3 cytoplasmic tails, which become packed into a more compact structure with reduced accesibility for trypsin. This is the Nck-binding or *locked* conformation. The reduced exposure of the CD3 cytoplasmic tails to trypsin must occur simultaneously with an increased exposure of the CD3ε PPS to Nck perhaps by fixing it into a conformation adequate for binding. We do not know the ultimate causes that explain why a trypsin-sensitive *loose* conformation in CD3ε is incompatible with a Nck-binding conformation of the PPS. We have however structural information (obtained by NMR) on how the PPS binds the SH3.1 domain of Nck (Borroto and Alarcón, in preparation). The CD3ε polypeptide makes an extensive fingerprint on the SH3.1 domain, where not only the central proline residues participate, but also upstream and downstream charged amino acids. This explains why CD3ε binds with abnormally high affinity for a SH3-ligand interaction (in the order of 0.1 µM), and may also indicate structural requirements for the interaction. In the *loose* conformation the sequences upstream and/or downstream of the PPS-and not necessarily the central prolines themselves-might be in non-binding conformation. The compaction of the CD3 tails resulting in the *locked* conformation may bring the Nck-binding sequence into the appropriate conformation.

In this study we have used anti-CD3 antibodies to elicit the conformational change that results in the trypsin-protected or *locked* conformation in both CD3ε and CD3ζ. The *locked* conformation was also induced when antibodies that recognize the variable domain of the TCRβ ectodomain were used for stimulation. We can therefore propose that a conformational change transmitted from the ectodomains of the TCRα/β heterodimer to the CD3 tails induces their *locked* conformation. We have attempted to demonstrate that a similar rearrangement takes place upon antigen stimulation. However these experiments have failed, probably due to the fact that continued pMHC-TCR interaction after lysis of the cells is necessary to preserve the conformational change [30].

The transmission of TCR conformational change across the plasma membrane presents a conceptual challenge. The structure of the CD3γ-CD3ε dimer ectodomains led to the proposal that the transmembrane domains of the dimer undergo a piston-like movement. In this way, the paired G beta strands of CD3ε and CD3γ (as well as those of CD3ε and CD3δ) would form a rigid rod-like connector that would displace the transmembrane helices [42]. On the other hand, monovalent Fab fragments of the anti-CD3 antibody OKT3 bind to CD3ε in a side-on orientation [43], and this interaction induces a conformational change in the TCR [17]. Considering this in conjunction with the electrostatic properties of CD3ε, it has been proposed that the transmission of the conformational change might require a rotational or scissor-like movement of both the transmembrane domains and the cytoplasmic tails of the CD3 subunits [43]. The protease-sensitivity data reported in the present paper support a model in which ligand binding to the TCRα/β heterodimers somehow promotes a combination of rotational and closing-scissor movements that simultaneously condense and shield the cytoplasmic tails of the CD3 subunits, while exposing key features, such as the PPS of CD3ε and ITAM A of CD3ζ. Studies with purified recombinant proteins have shown that all ITAM-bearing cytoplasmic tails studied (including those of the TCR and BCR) form oligomers in solution [44], although the interaction is weak (Kd in the order of 10 µM). The conformational change in the TCR might exploit this natural propensity of ITAM-bearing cytoplasmic tails to dimerize or oligomerize, by forcing the local effective ITAM concentration above the dissociation constant. Although the capacity of the three ITAMs of CD3ζ to homodimerize has not been measured on a one-by-one basis, it is tempting to speculate that the protease resistance of ITAM A could be caused by a higher propensity of this ITAM to dimerize in the resting TCR, compared to ITAMs B and C. Alternatively, protease resistance of ITAM A in the resting TCR could be due to the position of CD3ζ in the TCR complex, i.e. the tail of CD3ζ could occupy an internal position within the TCR complex, where the tails of the other CD3 subunits could shield the membrane most proximal region of CD3ζ's tail.

In our current model, the conformational change is initiated after binding of a cluster of two or more pMHC agonists to two or more TCRα/β heterodimers within a multivalent TCR complex [30]. This would generate a torque on the TCRα/β heterodimers that could be transmitted to the CD3 dimers. In turn, this binding would induce the rotation or sliding of the transmembrane domains of the CD3 dimer with respect to those of the TCRα/β heterodimers, and an ensuing transmission of this movement to the CD3 cytoplasmic tails. Crystal structures of TCRα/β ectodomains bound to pMHC complexes almost universally show that the orientation of TCRα/β is approximately diagonal to the MHC peptide-binding groove (for a review see [45]). We hypothesize that in the context of a multivalent TCR, the diagonal orientation imposed by pMHC binding on the two TCRα/β heterodimers may be responsible for the torque transmitted to the CD3 subunits.

## Materials and Methods

### Plasmids

The pGEX-4T1 derivative GST-SH3.1, containing the amino-terminal SH3 domain of Nckα, was kindly provided by Dr. R. Geha (Children's Hospital, Harvard Medical School, Boston). The pSRα-CD3ζ-Flag plasmid encoding human CD3ζ was generated by PCR. The truncated CD3ζA construct (pSRα-CD3ζA-Flag) expresses a protein truncated immediately after the first ITAM of human CD3ζ. Both constructs encode the Flag peptide fused to the C-terminus of the protein.

### Cells

The human T cell line Jurkat was maintained in complete RPMI 1640 supplemented with 10% fetal bovine serum (Sigma). The Jurkat cell clone fε-Jk expressing Flag-CD3ε has been described previously [17]. The JkζAF and JkζF cell lines were generated by stable transfection into Jurkat of the pSRα-CD3ζA-Flag and pSRα-CD3ζ-Flag, respectively.

### Antibodies

The anti-CD3ε mouse monoclonal antibody APA1/1 has been described previously [34], [35], [46], [47], as has the rabbit anti-CD3ζ antiserum 448 [48], [49]. The anti-CD3ζcit rabbit antiserum was obtained by repeatedly immunizing a white New Zealand rabbit with a purified peptide corresponding to the complete cytoplasmic tail of human CD3ζ, produced in *E.coli*. The anti-Cβ Jovi.1 mAb (IgG2a) and the anti-CD4 HP2/6 mAb (IgG2a) were a kind gift of Dr. M. Owen (CRUK, London, UK) and Dr. F. Sánchez-Madrid (Hospital de La Princesa, Madrid). Hybridomas producing the anti-CD3 mAb OKT3 (IgG2a), the anti-TCRβ mAb C305 (IgM) and the anti-CD8 OKT8 (IgG2a) were obtained from ATCC. Goat anti-CD3ε M20, mouse anti-human Vβ8 BV8 (IgG2b), mouse anti-hemagglutinin (HA) 12CA5 (IgG2a), and mouse anti-Flag (M2) antibodies were purchased from Santa Cruz Biotechnology, BD-Pharmingen, Roche and Sigma, respectively. Fab fragments were prepared using the Immunopure IgG1 Fab Preparation kit (Pierce) and confirmed by SDS-PAGE and silver staining.

### Stimulation and pull-down assay

10^7^ cells per point were collected, washed, resuspended in RPMI and incubated without serum at 37°C for 2 h. Cells were stimulated with 10 µg/ml soluble antibody for 5 min at 37°C and lysed in 20 mM Tris-HCl pH 8.2, 0.3% Brij96, 140 mM NaCl, 10 mM iodoacetamide and 2 mM EDTA (lysis buffer), plus 1 µg/ml each of leupeptin and aprotinin, and 1 mM phenyl methyl sulfonide fluoride (PMSF). For GST pull-down assays supernatants were first precleared with GST adsorbed to glutathione-Sepharose (Amersham Biosciences) before precipitation with GST-SH3.1 protein adsorbed to glutahione-Sepharose [17].

### Trypsin digestion

10^6^ cells per point were stimulated with 10 µg/ml soluble antibody for 5 min at 37°C and disrupted in lysis buffer without leupeptin, aprotinin or PMSF. A total of 1 µg/ml trypsin (Sigma) was added to the lysate and the reaction mix was incubated for 15 min at 37°C. Afterwards, 3× Laemmli's sample buffer was added, and samples boiled, to stop the enzyme activity. Alternatively, postnuclear cell lysates of unstimulated cells were incubated with the anti-CD3 antibody before trypsin digestion under the same conditions.

## Supporting Information

Supplemental Figure S1 TCR crosslinking with an antibody to a foreign CD3 epitope results in poor stimulation. (A) Anti-Flag stimulation of fepsilon-Jk cells is a weak inducer of the conformational change, but strong inducer of tyrosine phosphorylation. Jurkat T cells transfected with Flag-CD3epsilon (fepsilon-Jk cells) were stimulated with the indicated concentrations of an anti-Flag or anti-CD3 antibody (OKT3), lysed in Brij96, and TCR binding to GST-SH3.1 was revealed by immunoblotting with anti-CD3zeta antibody. (B) Compared to anti-CD3, the anti-Flag antibody poorly activates T cells. The expression of CD69 and CD25 was examined in fepsilon-Jk cells 24 h after stimulation with OKT3 (closed symbol) or anti-Flag (open symbol). Induction of programmed cell death was examined 48 h after stimulation with immobilized antibodies by propidium iodide exclusion (PI staining) or by cell cycle analysis (% cells in sub-G1).
(0.84 MB TIF)

Supplemental Figure S2 Expected and observed sizes of partial trypsin digestion of the CD3zeta tail. A cartoon of the CD3epsilon and CD3zeta subunits showing potential trypsin cleavage sites (arrows). Transmembrane domains are indicated with grey boxes, and the relative positions of the three ITAMs are marked. The observed sizes of the partial digestion products were calculated from results shown in Fig. 1C.
(3.46 MB TIF)
