# Supplementary figures and images for: T Cell Receptor Engagement Triggers Its CD3ε and CD3ζ Subunits to Adopt a Compact, Locked Conformation
Source: PLoS One. 2008 Mar 5;3(3):e1747. doi: 10.1371/journal.pone.0001747 (PMC2254190; doi:10.1371/journal.pone.0001747)

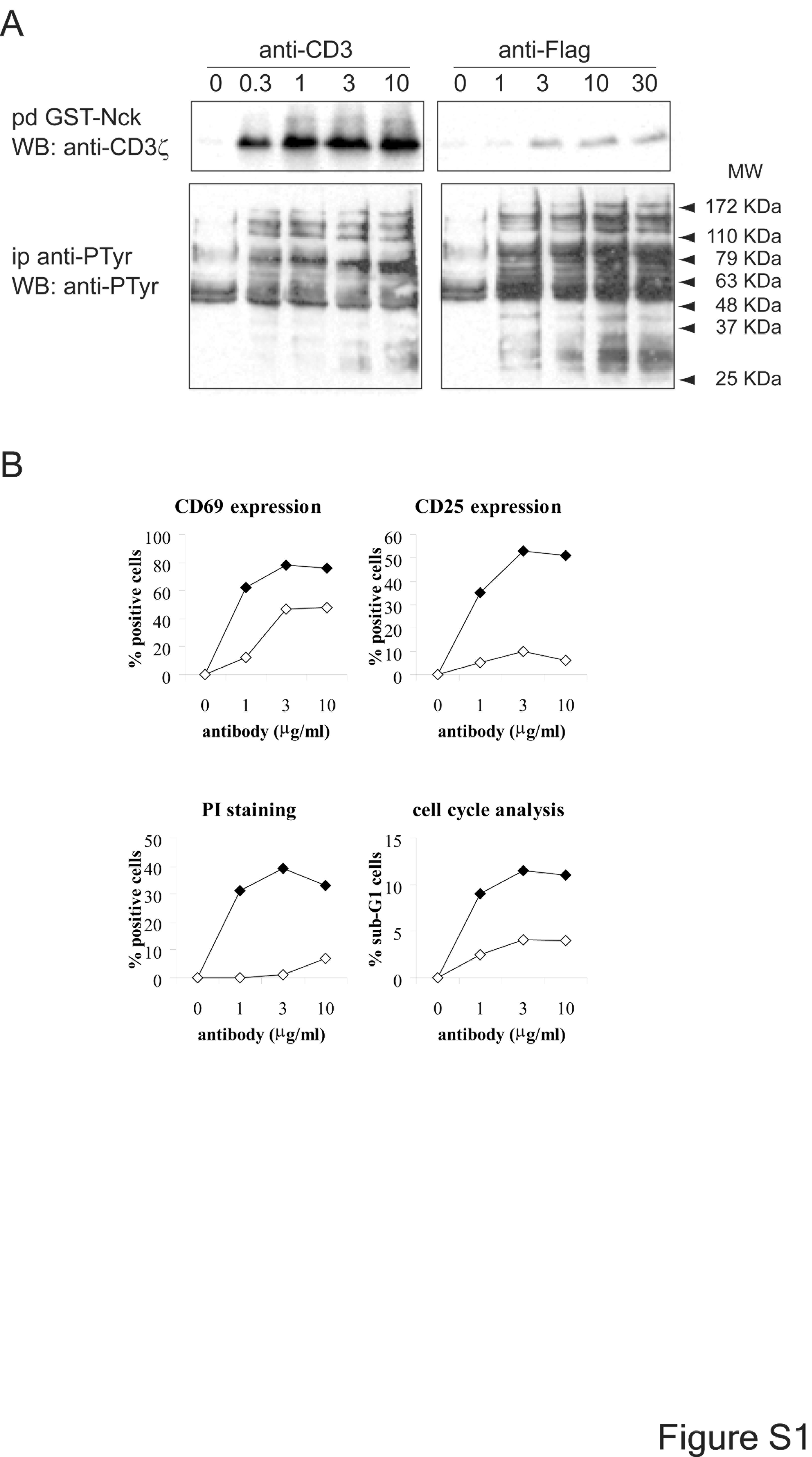

Supplement: Supplemental Figure S1 — TCR crosslinking with an antibody to a foreign CD3 epitope results in poor stimulation. (A) Anti-Flag stimulation of fepsilon-Jk cells is a weak inducer of the conformational change, but strong inducer of tyrosine phosphorylation. Jurkat T cells transfected with Flag-CD3epsilon (fepsilon-Jk cells) were stimulated with the indicated concentrations of an anti-Flag or anti-CD3 antibody (OKT3), lysed in Brij96, and TCR binding to GST-SH3.1 was revealed by immunoblotting with anti-CD3zeta antibody. (B) Compared to anti-CD3, the anti-Flag antibody poorly activates T cells. The expression of CD69 and CD25 was examined in fepsilon-Jk cells 24 h after stimulation with OKT3 (closed symbol) or anti-Flag (open symbol). Induction of programmed cell death was examined 48 h after stimulation with immobilized antibodies by propidium iodide exclusion (PI staining) or by cell cycle analysis (% cells in sub-G1). (0.84 MB TIF) [file pone.0001747.s001.tif]

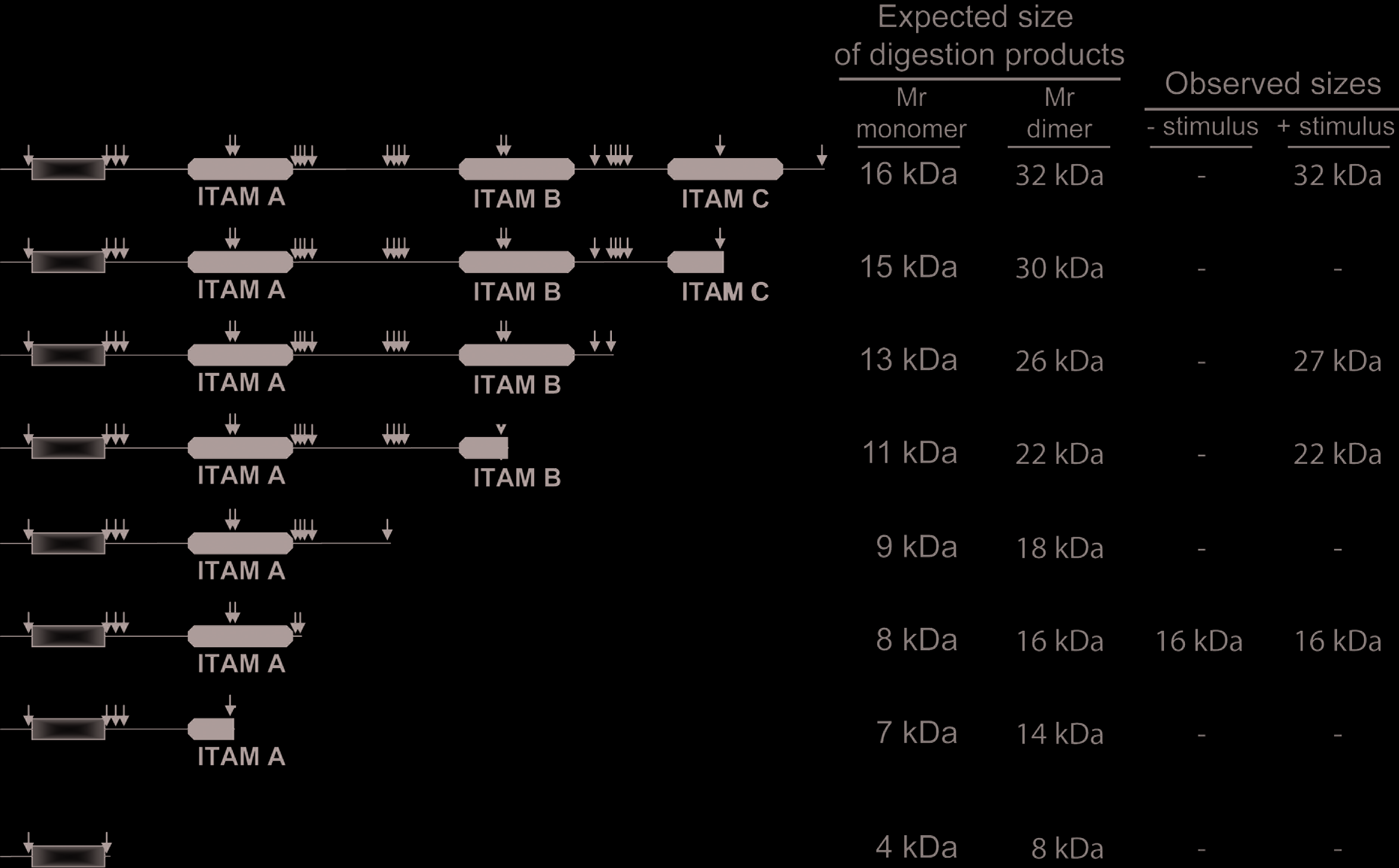

Supplement: Supplemental Figure S2 — Expected and observed sizes of partial trypsin digestion of the CD3zeta tail. A cartoon of the CD3epsilon and CD3zeta subunits showing potential trypsin cleavage sites (arrows). Transmembrane domains are indicated with grey boxes, and the relative positions of the three ITAMs are marked. The observed sizes of the partial digestion products were calculated from results shown in Fig. 1C. (3.46 MB TIF) [file pone.0001747.s002.tif]
